# Microbial lectome versus host glycolipidome: How pathogens exploit glycosphingolipids to invade, dupe or kill

**DOI:** 10.3389/fmicb.2022.958653

**Published:** 2022-08-19

**Authors:** Anna Bereznicka, Krzysztof Mikolajczyk, Marcin Czerwinski, Radoslaw Kaczmarek

**Affiliations:** Department of Immunochemistry, Laboratory of Glycobiology, Hirszfeld Institute of Immunology and Experimental Therapy Polish Academy of Sciences, Wrocław, Poland

**Keywords:** glycosphingolipids, bacteria, viruses, fungi, blood groups antigens

## Abstract

Glycosphingolipids (GSLs) are ubiquitous components of the cell membranes, found across several kingdoms of life, from bacteria to mammals, including humans. GSLs are a subclass of major glycolipids occurring in animal lipid membranes in clusters named “lipid rafts.” The most crucial functions of GSLs include signal transduction and regulation as well as participation in cell proliferation. Despite the mainstream view that pathogens rely on protein–protein interactions to survive and thrive in their hosts, many also target the host lipids. In particular, multiple pathogens produce adhesion molecules or toxins that bind GSLs. Attachment of pathogens to cell surface receptors is the initial step in infections. Many mammalian pathogens have evolved to recognize GSL-derived receptors. Animal glycosphingolipidomes consist of multiple types of GSLs differing in terminal glycan and ceramide structures in a cell or tissue-specific manner. Interspecies differences in GSLs dictate host specificity as well as cell and tissue tropisms. Evolutionary pressure exerted by pathogens on their hosts drives changes in cell surface glycoconjugates, including GSLs, and has produced a vast number of molecules and interaction mechanisms. Despite that abundance, the role of GSLs as pathogen receptors has been largely overlooked or only cursorily discussed. In this review, we take a closer look at GSLs and their role in the recognition, cellular entry, and toxicity of multiple bacterial, viral and fungal pathogens.

## Introduction

**Figure 1:**
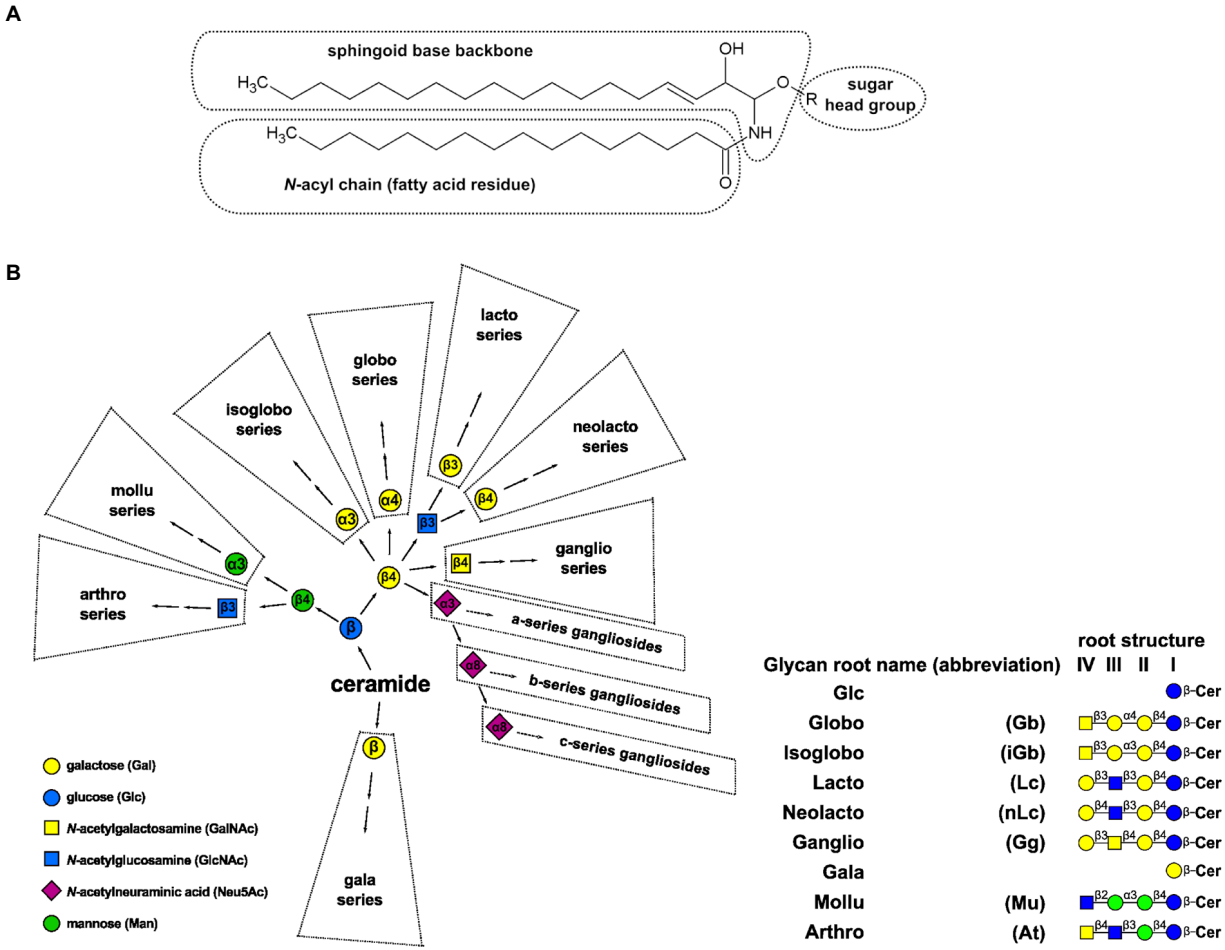
**(A)** Schematic representation of a GSL structure. Ceramide moiety comprises sphingoid backbone (based on sphingosine d18:1^Δ4^) and N-acyl chain. **(B)** A backbone relational depiction of major GSL synthesis pathways and an overview of glycan root structures. Names and symbols are presented on figure according to IUPAC (Merrill et al., 2007), while monosaccharide symbols are consistent with Varki and Sharon recommendations (Varki et al., 2017); Cer: ceramide.

Glycosphingolipids (GSLs) are amphipathic lipids consisting of hydrophilic glycan and hydrophobic ceramide (N-acylsphingosine) moieties. Ceramides comprise a sphingoid base (aminoalcohol) linked to a fatty acid through an amide bond (Figure 1A). Due to the variety of sugar structures (ranging from monosaccharide residues to branched glycan moieties), sphingoid and acyl chains, GSLs represent the largest group of sphingolipids (Merrill et al., 2007; Gault et al., 2010). These molecules sort with specific microdomains in the plasma membrane called lipid rafts. GSLs are classified into broad types based on carbohydrate composition. In animals, glucosylceramide is the cornerstone for the synthesis of all complex GSLs, with the largest containing over 20 sugar residues (found in the human placenta; Ladisch et al., 1995; Merrill et al., 2007; Ishibashi et al., 2009). Step-wise extension of these glycan moieties generates a series of neutral root structures that are commonly found in different species and tissues, including globo, isoglobo, lacto, neolacto, ganglio, gala, muco, arthro, and mollu (Figure 1B). These root structures serve as the basis for a widely used nomenclature system (in addition to traditional names). For example, a GSL blood group antigen historically called Forssman, which belongs to the globo series, is a globotetraosylceramide with *α*1,3-linked *N*-acetylgalactosamine or IV^3^-α-GalNAc-Gb4Cer (with the roman numeral indicating which sugar in the root structure, counting from the ceramide end, carries the substituent and the superscript indicating the linkage position on that sugar, in this case, C-3; Chester, 1997; Merrill et al., 2007). Traditionally, all acidic GSLs containing one or more sialic acid residues are called gangliosides, including the compounds based on other than ganglio root structures (Chester, 1997; Yu et al., 2009; Giussani et al., 2014). Lactosylceramide may also be directly mono-, di- or trisialylated, whence elongation of the ganglio root structure continues in the same way as for the unsialylated lactosylceramide, generating the 0-, a-, b- and c-series pathways of ganglioside synthesis, respectively (Merrill et al., 2007; Giussani et al., 2014).

Many viruses, bacteria and fungi produce adhesins or toxins that recognize GSLs. Cell surface GSLs provide pathogens with unique opportunities to invade and thrive in the host. Adhesins and toxins specifically recognize oligosaccharide chains of GSLs, which protrude beyond the cell membrane, but subsequent events and the fate of the microbe or toxin may depend on additional factors, such as density of GSL packaging in the host cell membrane, the cell membrane microenvironment, and other membrane components, including proteins, mostly transmembrane or GPI-anchored (Duncan et al., 2002; Taïeb et al., 2004). High-affinity binding of pathogens to GSL receptors involved in crucial functions in host cells such as signal transduction pathways or intracellular transport have not been thoroughly studied to date. The affinity linkage between microbial toxins or adhesins to host GSL receptors may affect the mechanism of toxicity or adhesion and the intracellular transport of pathogens or their virulence factors. The hydrophobic part of GSLs affects the conformation of the glycone part, and hence, it may affect the receptor functions of GSLs (Smith et al., 2004; D’Angelo et al., 2013). Recent studies have shown that the molecular ‘spacer’ function of cholesterol in lipid membranes could also affect the conformation and binding properties of GSLs. Some bacterial virulence factors (e.g., cholera toxins) bind to receptors located in condensed complexes with cholesterol in lipid membranes *in vitro* (Radhakrishnan et al., 2000).

Many pathogens recognize sugar antigens (Unione et al., 2017; Thompson et al., 2019), which has driven modifications of cell surface sugars by hosts as a means of defense. To hinder recognition of cell surface sugars by pathogens, hosts use three strategies:

1. The sugar antigen recognized by the pathogen is modified so that it is no longer recognized by the pathogen (Cserti and Dzik, 2007);
2. The sugar antigen recognized by the pathogen ceases to be produced altogether, e.g., *via* pseudogenization. One example is the disappearance of the Galα1 → 3Gal antigen, which, additionally, increases resistance to pathogens carrying that structure (Koike et al., 2002);
3. The sugar antigen becomes a decoy receptor that binds the pathogen but prevents it from entering. Examples include the A and B blood group antigens and the cholera toxin or sugar antigens from the human milk (A/B, Lewis) and rotaviruses (Anstee, 2010; El-Hawiet et al., 2015; Orczyk-Pawiłowicz and Lis-Kuberka, 2020).

**Figure 2:**
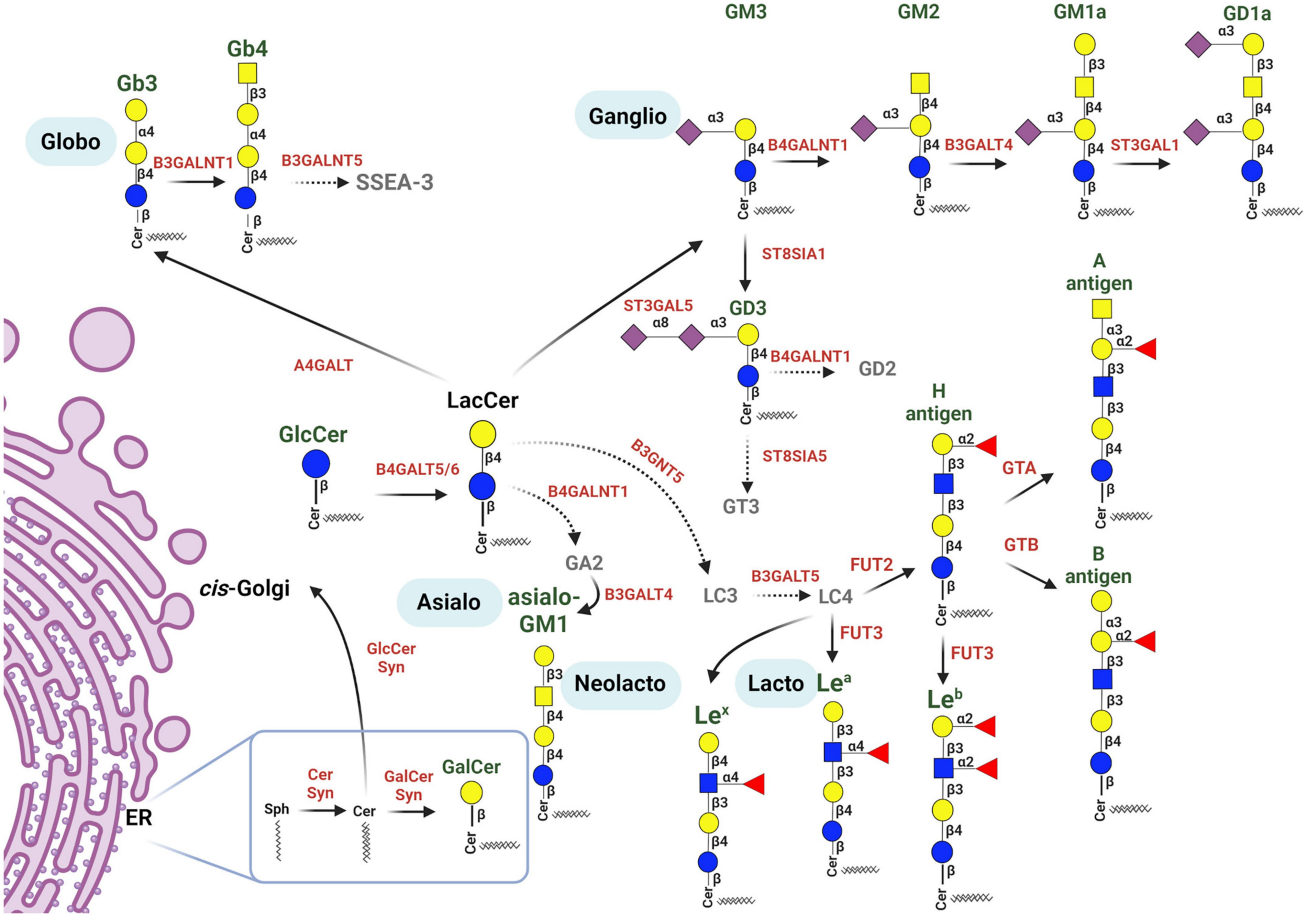
Schematic representation of major glycosphingolipid biosynthesis pathways, highlighting GSLs participating in pathogen binding. Biosynthesis of GSLs proceeds in the endomembrane system, beginning in the endoplasmic reticulum by ceramide synthesis from sphingoid base and acyl-CoA, and continues in the Golgi apparatus. Ceramide may be galactosylated in the ER (producing GalCer) or transported to the *cis*-Golgi to produce GlcCer and LacCer. LacCer is used for the production of more complex GSLs by specific Golgi-resident glycosyltransferases, creating several GSL series pathways. LacCer is utilized by (1) *β*1,4-*N*-acetylgalactosylaminyltransferase (B4GALNT1) initiating the asialo series; (2) β1,3-*N*-acetylglucosaminyltransferase (B3GNT5), producing Lc3, which is converted to Lc4, the precursor for the lacto and neolacto series GSLs; (3) α1,4-galactosyltransferase (A4GALT), producing Gb3, thus initiating the globo series pathway; (4) α2,3-sialyltransferase (ST3GAL5), producing GM3, which belongs to the ganglio series. The ABO histo-blood group antigens are subsequently formed from the H antigen (it is also the precursor of the Le^b^ antigen, belonging to the Lewis blood group system) by specific GTs; α1,3-*N*-acetylgalactosyltransferase (A transferase) synthesizes the A antigen, while α1,3-galactosyltransferase (B transferase) synthesizes the B antigen. The Le^a^ and Le^x^ blood group antigens are created by fucosyltransferase 3. More complex glycan structures of ganglio-series GSLs are formed by a sequential action of different GTs, such as *N*-acetylgalactosaminyltransferases, galactosyltransferases and sialyltransferases, producing the GM2, GM1a and GD1a gangliosides. GM3 may be further processed by α2,8-sialyltransferase 1, forming GD3. GSLs in green play roles in interactions with pathogens. Glycosyltransferases involved in GSL synthesis are shown in red [Figure was created with BioRender.com].

**Figure 3:**
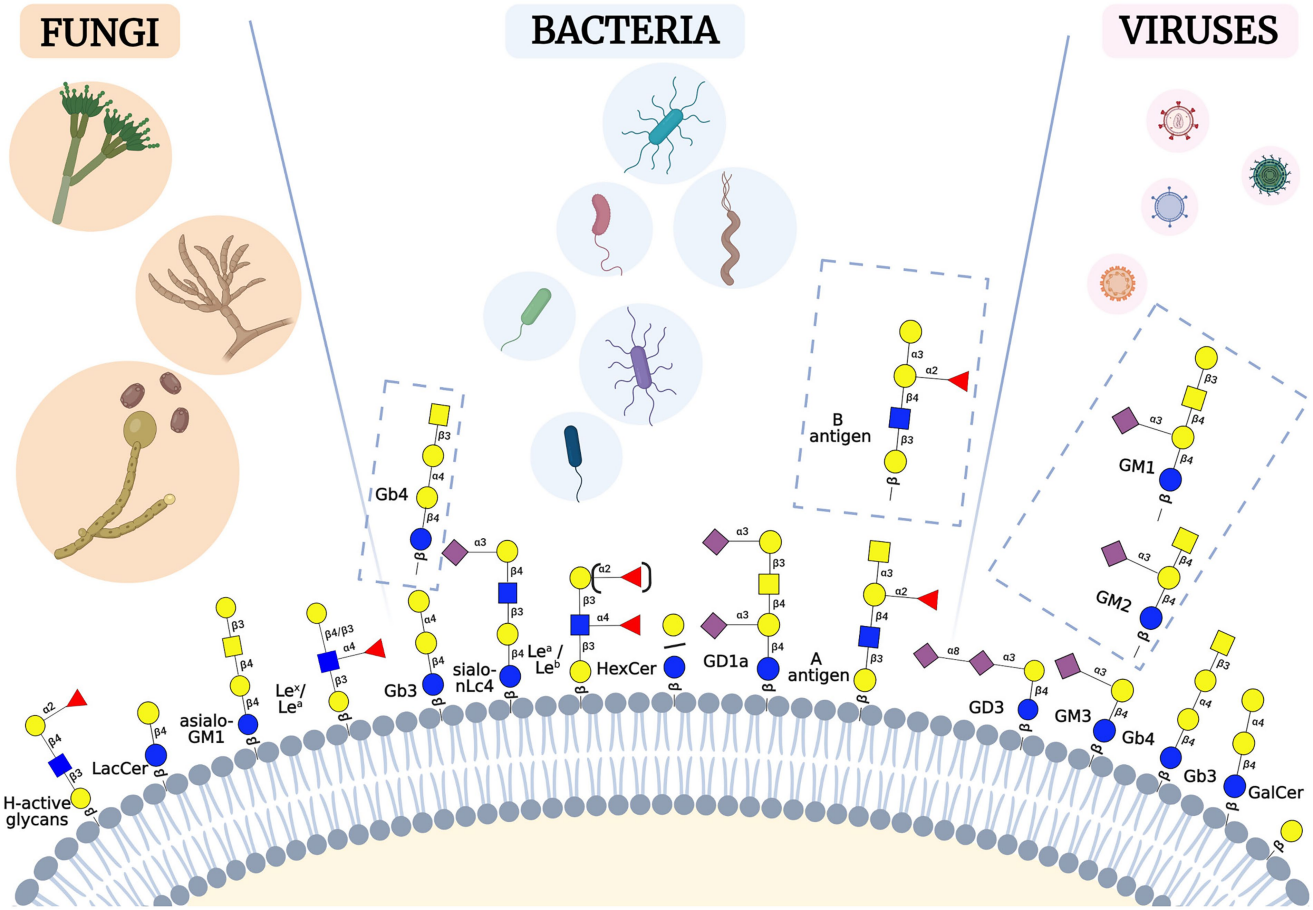
Schematic representation of selected GSLs involved in host-pathogen interactions. Fungi, bacteria and viruses may bind various types of GSLs residing in the human membranes. These GSLs belong to different GSL-series, such as asialo, lacto, neolacto, ganglio [Figure was created with BioRender.com].

Many GSL blood group antigens may act as a pathogen or toxin receptors or co-receptors. Here, we review bacterial, viral, and fungal pathogens or toxins, and the mechanisms whereby they hijack cell surface GSLs to invade, evade or incapacitate the host cells (summarized in Figures 2, 3; Table 1).

**Table 1:** Summary of described pathogen ligands, host receptors and relevance of these interactions in infectious diseases epidemiology.

Pathogen	Pathogen ligand (s)	GSLs receptor(s)	Possible implication/Epidemiology	References
BACTERIA				
*Acinetobacter baumannii*	OmpA, Bap, BLP-1 and BLP-2	GalCer, GlcCer, GgO_3_, GgO_4_, Lc3, nLc4, iGb3	no binding to glycoconjugates with terminal Galα1 → 4Gal or Galα1 → 3Gal moieties (besides iGb3); resistant to almost all available antimicrobials approximately 1 million cases annually	Logan et al., 2018; Madar Johansson et al., 2020
*Campylobacter jejuni* enteritis	LPS	GM1, GD1a	bacteria produce antibodies against host GM1 and GD1a resulting in Guillain-Barré syndrome development.	Yuki, 1997; Cawthraw et al., 2002
*Clostridium botulinum*; *Clostridium tetani*; *Clostridium difficile*	Botulinum neurotoxin; tetanus neurotoxin (TeNT); *Clostridium difficile* toxin (CDT)	C-type: sialoparagloboside, GM3 and Lc3Cer; Type A: Lc3Cer, Gb3 and LacCer GM1a, GD1a, GT2, GD3; GM1	25% of an average of 110 reported cases of botulism annually in the US are foodborne botulism 500–600 cases annually before a vaccine at present, 8 tetanus toxoid-containing vaccines are administrated. Recommended in a different range of age epidemiologic surveillance efforts have been directed toward mitigating hospital-acquired *C. difficile* (most of the diagnosed infection), over 450,000 cases per year (the USA, 2017)	Montecucco, 1986; Yowler and Schengrund, 2004; CDC Botulism | Epidemiological Overview for Clinicians, 2019 Chen et al., 2008, 2009; Pinkbook: Tetanus | CDC, 2021 Orrell et al., 2017; Fu et al., 2021
Shiga toxin-producing *Escherichia coli* (STEC) strains Uropathogenic *E.coli* (UPEC) strains Enterotoxigenic *Escherichia coli* (ETEC) strains	Shiga toxins (subunits B); PapG subunits, SfaH and FocH Fimbrial structures are referred to as colonization factors	Gb3 (main receptor), Gb4, Forssman (mostly Stx2e in piglets, also identified in pigeons); P antigen sialylα2 → 3lactose, GalNAcβ1 → 4Galβ disaccharide in asialoceramides iGb3, nLc4, GgO_3_, GgO_4_, LacCer, HBGAS	Gb3-depleted cells (*A4GALT* gene knock-out) become insensitive to Shiga toxins; p phenotype individuals resistant to Shiga toxins toxicity; over 63, 000 cases (USA); huge economic losses during outbreaks 11, 000, 000 cases/year in the USA, approximately 150, 000, 000 worldwide. Several factors are related with develop a UTI phenotype: dysfunctions of the urinary tract and/or genetic mechanisms involved in the innate immune response control 220, 000, 000 cases and over 50, 000 deaths annually (in developing countries among children aged <5 years)	Okuda et al., 2006; Bruyand et al., 2018; Fatima and Aziz, 2022 Söderhäll et al., 1997; Khan et al., 2000a; Kaczmarek et al., 2014; Terlizzi et al., 2017 Jansson et al., 2006; Buuck et al., 2016–2017
*Helicobacter pylori*	BabA	Lewis antigens (Le^b^), LacCer	individuals are more susceptible to the binding of bacterial adhesin; in the mouse model Le^b^ plays a crucial role in *H. pylori* mucosal attachment, in man, no relationship between *H. pylori* and host Lewis status has been found; the most common gastrointestinal tract bacterial infection worldwide (it was estimated that half of the human population is infected by *H. pylori*)	Angström et al., 1998; Benktander et al., 2012; Epidemiology of *Helicobacter pylori* - Sonnenberg - 2022 - Alimentary Pharmacology & Therapeutics - Wiley Online Library
*Pseudomonas aeruginosa*	LecA	Gb3	interaction with hosts receptor as “lipid zipper” Lec-A/Gb3 ligand suppression results in a reduced invasion of *Pseudomonas aeruginosa* and is a promising strategy for drug development *P. aeruginosa* was the most common cause of ventilator-associated pneumonia globally, accounting for 26% of cases	Blanchard et al., 2008; Kuhaudomlarp et al., 2020; Reynolds and Kollef, 2021
*Vibro cholerae*	Cholera toxin (CT)	GM1	cholera affects impoverished populations without proper access to adequate water and sanitation (mostly in Asian countries) The global oral cholera vaccine was created in 2011 for a rapid response to cholera outbreaks and protects against cholera	Ramamurthy et al., 2019; Deen et al., 2020
VIRUSES				
HCMV	N/A	SGGLs, especially SGLPG	lactosamine repeats could also play a role in the preferential binding of HCMV; SGLPG had a stronger inhibitory effect; 68.7% among HCMV positive children are aged <6 months, and children aged above 6 months result in 31.3%	Ogawa-Goto et al., 1998; Li et al., 2020
HIV	gp120	Gb3, GM3, GD3	soluble Gb3 (adamantylGb3) analogue may bind gp120 and thus inhibit viral infection *in vitro* gp120 binding to GalCer triggers events that allow infecting CD4-negative cells. Modification of the lipid moiety in GalCer affects binding of the virus to CD4 +/− cells with varying degrees of inhibition the inducible modulation of GSL content on the host cell surface, especially under proinflammatory conditions, can have a significant impact on HIV interacts with the host cell (influencing the composition of the plasma membrane from which *de novo* virions will bud) approximately 37.7 million people living with HIV (data from 2020), over two-thirds of whom (25.4 million) are in the WHO African Region	Rawat et al., 2005; Puryear and Gummuluru, 2012; Aigal et al., 2015; HIV/AIDS, 2021
Human Parvovirus B19	Interaction with VP1u, but direct ligand unknown	P antigen (additional receptor α5β1 integrin and Ku80 autoantigen)	presence of Gb4 on the host cell surface is required, but not sufficient for productive infection most common in school-aged children; transmission of the virus occurs through respiratory secretions and blood products	Norja et al., 2012; Bieri et al., 2021; Macri and Crane, 2022
Noroviruses	VLPs, receptor binding site lies at the outermost end of the P domain of capsid	HBGAS (ABH, Lewis)	P domain dimer plays a crucial role in the formation of the receptor binding interaction noroviruses can bind to non-HBGAS receptors, e.g.: heparan sulfate, histon 1, or breast milk glycans HMO can inhibit noroviruses infections noroviruses infections are the most common foodborne illnesses (according to CDC) 21 million cases of gastrointestinal illness in the USA each year	Almand et al., 2017; Koromyslova et al., 2017; Chassaing et al., 2020; Capece and Gignac, 2022; Norovirus Worldwide | CDC, 2022
Polyomaviruses	BKV and JCV polyomaviruses Merkel Cell Polyomavirus (VP1 protein) SV40 virus	NeuAcα2→3Gal and NeuAcα2→6Gal on gangliosides; GT1b GM1 (with Neu5Gc)	pseudoreceptors are O-l-linked glycoproteins α4β1 integrin may serve as co-receptor virus entering the host cell by clathrin-dependent endocytosis HIV/AIDS patients have been reported more susceptible to polyomaviruses infections	Ahsan, 2006; Erickson et al., 2009; Hurdiss et al., 2018; Zhou et al., 2020
Rotaviruses	VP8 (spike protein)	HBGAS (A, H, Le^b^)	RTs infections cause significant economic losses in agriculture over 500,000 deaths in developing countries could possess a frequent cause of childhood morbidity in industrialized countries more than 600,000 young children die from RTS infections approximately 2.4 million children are hospitalized annually from rotavirus disease (mostly in South-East Asia and sub-Saharan Africa)	Delorme et al., 2001; Mukherjee and Chawla-Sarkar, 2011; Barbé et al., 2018; Thompson et al., 2019
FUNGI				
*Candida spp.* (*C. albicans* *C. glabrata* *C. parapsilosis* *C. tropicalis* *C. krusei*)	β-(1,3)-glucan, β-(1,6)-side chain branched glucan, fucose-binding lectins	Lactosylceramide, asialo-GM1, Le^a^, Le^x^ and H-active glycans ^1^	cause candidiasis, including vaginal (only for *C. albicans*), 700,000 cases annually worldwide Le(a-b-) phenotype was connected with recurrent vaginitis in women nonsecretors are more prone to *C. albicans* FUT2-null mice showed a threefold increased sensitivity for infections with *C. albicans* *C. albicans* most frequently colonized patients with the O blood group, with a higher incidence of nonsecretor status among the patients with peptic ulcer	Critchley and Douglas, 1987; Jimenez-Lucho et al., 1990; Brassart et al., 1991; Tosh and Douglas, 1992; Burford-Mason et al., 1993; Cameron and Douglas, 1996; Johansson et al., 2000; Sato et al., 2006; Bryan et al., 2015; Tafesse et al., 2015; Bongomin et al., 2017
*Cryptococcus neoformans*	Hyaluronic acid (HBMEC cells)	Lactosylceramide	cause cryptococcosis, 220,000 cases annually worldwide (for people living with HIV/AIDS) liposomes containing LacCer inhibit the binding of this fungi the binding of *C. neoformans* to LacCer is affected by the structure of ceramide moiety terminal galactose residue of LacCer is essential for binding and its removal abolishes binding	Jimenez-Lucho et al., 1990; Jong et al., 2007, 2012; May et al., 2016; Florek et al., 2021
*Histoplasma capsulatum*	HSP60, β-(1,3)-glucan	Lactosylceramide, GM1	caused histoplasmosis, 500,000 cases every year in the U.S. the interaction between *H. capsulatum* and macrophages required LacCer and GM1, and lipid rafts mediated in pathogen internalization GM1 may be a co-receptor in the initial steps of *H. capsulatum* binding	Jimenez-Lucho et al., 1990; Lin et al., 2010; Benedict and Mody, 2016; Guimarães et al., 2019
*Paracoccidioides brasiliensis*	N/A	GalCer, LacCer, CTH, GD3, GD1a GM1, GM3, *N*-acetylneuraminic acid ^2^	responsible for paracoccidioidomycosis, 15,000 cases detected since 1930, mostly in Brazil GM1 localized in lipid rafts of epithelial cells and lung fibroblasts may serve as receptor for *P. brasiliensis* removal of *N*-acetylneuraminic acid from human alveolar cells resulted in decreased adhesion of *P. brasiliensis* conidia	Jimenez-Lucho et al., 1990; González et al., 2005; Maza et al., 2008; Ywazaki et al., 2011
*Pneumocystis jiroveci*	β-(1,3)-glucan	Lactosylceramide	responsible for pneumonia in immunocompromised patients current global estimates are as high as 500,000 annual cases, with a mortality of 10 to 30% LacCer of host cells involves in binding and internalization of *P. jirovici*	Bongomin et al., 2017; Chan et al., 2021; Kolbrink et al., 2022
*Saccharomyces cerevisiae*	β-(1,3)-glucan	Lactosylceramide	commensal, although may cause opportunistic infections in patients with chronic disease, cancer, and immunosuppression	Jimenez-Lucho et al., 1990; Zimmerman et al., 1998
*Sporothrix schenckii*	N/A	Lactosylceramide	responsible for sporotrichosis in the state of Rio de Janeiro, Brazil, more than 2,200 cases were reported during 1998–2009 another study suggested a rate of 48 to 60 sporotrichosis cases per 100,000 population in the south-central highlands of Peru	Jimenez-Lucho et al., 1990; Pappas et al., 2000; Barros et al., 2011

N/A, no data, HBMEC: Human brain microvascular endothelial cells, HBGASs: Human blood group antigens.

1 The administration of H-active glycans in the case of *C. albicans* vaginitis infection, could decrease the sensitivity for this pathogen.

2 Examined only for *P. brasiliensis* conidia.

## Interaction between bacteria and host GSLs

### Acinetobacter baumannii

*Acinetobacter baumannii* is a nosocomial pathogen typically causing pneumonia, but also urinary tract infections, skin infections and meningitis, mostly in hospitalized patients (Madar Johansson et al., 2020). Multiresistant strains of *A. baumannii* are a growing public health issue and they are on WHO’s high priority list of pathogens demanding urgency in development of new antimicrobial compounds. The outer membrane protein A (OmpA), the biofilm-associated protein (BAP), the *Acinetobacter* trimeric autotransporter adhesin (Ata) and the BAP-like proteins 1 and 2 (BLP-1 and BLP-2) were shown to take part in adhesion of bacterial cells to human epithelial cells and biofilm formation (Giannouli et al., 2013). Mass spectroscopy analysis of human small intestine glycosphingolipids recognized by *A. baumannii* proteins revealed lactotetraosylceramide (Galβ3GlcNAcβ3Galβ4Glcβ1Cer) and neolactotetraosylceramide (Galβ4GlcNAcβ3Galβ4Glcβ1Cer). The bacteria are also bound to lactotriaosylceramide (GlcNAcβ3Galβ4Glcβ1Cer), isoglobotriaosylceramide, gangliotriaosylceramide, gangliotetraosylceramide, galactosylceramide, lactosylceramide with phytosphingosine and/or hydroxy-fatty acids, indicating a *N*-acetylglucosamine moiety as a basic structure recognized by *A. baumannii* (Madar Johansson et al., 2020). Despite the high degree of homology with *P. aeruginosa*, LecA protein (PA-IL), LecA of *A. baumannii* does not bind to glycoconjugates with terminal Galα1 → 4Gal or Galα1 → 3Gal moieties (besides iGb3; Blanchard et al., 2008; Madar Johansson et al., 2020).

### *Campylobacter jejuni* enteritis

Neurons of human peripheral nerves produce large amounts of gangliosides GM1 and GD1a. Strikingly the high level of expression of GM1 on the nodal membranes of human motor nerves has substantial implications for the persistence and severity of post-infectious complications (Yanaka et al., 2019). Most of the described strains of *C. jejuni* can also produce glycoconjugates (GM1-like or GD1a-like lipooligosaccharides) on the bacterial cell surface. Anti-GM1 IgG1 autoantibodies arising in response to *C. jejuni* infections may lead to complement-mediated motor nerve injury. The binding of anti-GM1 autoantibodies to peripheral motor nerves underlies Guillain–Barré syndrome (GBS), which is defined as a post-infectious autoimmune neuropathy with characteristic acute limb weakness (Lee et al., 2004; Prendergast et al., 2004). GBS follows *C. jejuni* infections in one-third of patients (Yamana et al., 2019). Moreover, GBS was identified in patients previously infected with: *Mycoplasma pneumoniae*, herpes simplex virus type 1 (HSV-1), and Epstein–Barr virus (Yuki, 1997; Sheikh et al., 1998; Tse et al., 2012).

### *Clostridium* sp.

*Clostridium botulinum* produces seven serotypes of Botulinum neurotoxin (A-G), each of which is a single polypeptide of approximately 150 kDa consisting of two heavy and light chains linked by a disulfide bond. The carboxy part of the heavy chain participates in adhesion, while the N-terminal part mediates entry of the light chain (zinc endoprotease) into the host cell (Orrell et al., 2017). The toxin binds to its receptors on the terminal neurons of neuromuscular junctions. A number of studies indicate that the 16S and 19S complexes of the hemagglutinin complex may bind to specific GSLs. Thus, the C-type progenitor binds sialoparagloboside, ganglioside GM3 and paragloboside. The Type A progenitor recognizes GSLs with terminal galactose, particularly paragloboside and Gb3 and LacCer. Many studies indicate that a large number of BoNT serotypes require the presence of 1b-series gangliosides for the most effective binding to the target cell. The only exception is serotype G, which binds in the absence of gangliosides. Detailed studies have shown that ganglioside binding occurs through the C-terminus of a heavy chain, and removal of ten amino acid residues from its sequence abolishes the binding (Barth et al., 2004; Yowler and Schengrund, 2004).

Another strain of *Clostridium* sp., *C. tetani* produces the tetanus neurotoxin (TeNT). The toxin binds to vesicle-associated membrane protein-2 (VAMP-2), which inhibits neurotransmitter release in the central nervous system, and thus causes spastic paralysis and death. The molecular mechanism of TeNT entry into neurons is still unclear. Receptor binding occurs on presynaptic membrane of human *α*-motor neurons and involves gangliosides (Montecucco, 1986; Mocchetti, 2005). Interactions between TeNT and gangliosides have been well described in structural and biochemical studies *in vitro* and *in vivo*. Such interactions on the host cell surface required two molecules of gangliosides: an a-series ganglioside (GD1a, involving its lactose moiety and the TeNT W carbohydrate-binding pocket) and a b-series ganglioside (GT2, GD3, involving their sialic acids moieties and the TeNT R carbohydrate-binding pocket; Chen et al., 2009). TeNT cytotoxicity requires both receptors (Rummel et al., 2003; Chen et al., 2008, 2009).

*Clostridium difficile* toxin (CDT) consists of an enzymatic component with ADP-ribosyltransferase activity and a translocation/binding component. This binary actin-ADP-ribosylating toxin can depolymerize the actin cytoskeleton and induce formation of microtubule-based membrane protrusions (Barbieri et al., 2002). These activities enhance bacterial adhesion and colonization by virulent strains of *C. difficile*. The main glycosphingolipid receptor for this toxin is the ganglioside GM1 localized in lipid microdomains. Numerous studies have shown that composition of the lipid bilayer microenvironment, especially the presence of cholesterol, can favor membrane protrusions. Indeed, depletion of cholesterol from lipid microdomains abolishes the effects of the toxin and reduces membrane protrusions. The microenvironment of plasma membrane and composition of lipid bilayer are the main players in ligand-receptor interactions (Barth et al., 2004; Schwan et al., 2011).

### Escherichia coli

Uropathogenic *E.coli* (UPEC) strains produce two types of adhesive surface organelles named Chaperone-usher (CU) fimbriae 1 and P, found in many Gram-negative bacteria (Wurpel et al., 2013). FimH, the adhesive subunit of type 1 fimbriae, binds uroplakins, which are mannosylated glycoproteins present on the bladder epithelial cells (urothelium). This is a key step in the invasion of urothelium during chronic *E. coli* cystitis (Mulvey et al., 1998). Type P fimbriae contain PapG subunits, which recognize Gal*α*1 → 4Gal*β* residues in the globo-series GSLs on kidney epithelium. Some of these GSLs are histo-blood group antigens belonging to the P1PK blood group system formerly known as P, which is why the P fimbriae were so named (Söderhäll et al., 1997; Kaczmarek et al., 2014; Terlizzi et al., 2017). Pili F1C and S adhesins: SfaH and FocH bind sialylα2 → 3lactose and GalNAcβ1 → 4Galβ disaccharide in asialoceramides (Khan et al., 2000a,b). The Fm1H is an adhesin of F9 and binds Galβ1 → 3GalNAc (the TF antigen). Studies have shown that replacing 3 amino acids in 3 loops forming FimH and Fm1H caused a change in binding specificity of these adhesins from terminal *α*-D mannose to terminal β-D galactose or β-D GalNAc (Moonens and Remaut, 2017).

Enterotoxigenic *Escherichia coli* (ETEC) strains are the main cause of diarrhea in young children from developing countries. Adhesion and colonization of host intestinal epithelium depend on heat-stable and/or heat-labile enterotoxins. Fimbrial structures on bacterial cells’ surfaces are referred to as colonization factors (CFs) that mediate the adhesion of ETEC. Recently published studies indicate that ETEC can bind to numerous GSLs structures on host intestinal epithelium, e.g.: iGb3, nLc4, GgO3, GgO4, lactosylceramide with phytosphingosine and/or hydroxy fatty acids and glycosphingolipids with terminal HBGA determinants (Le^a^, Le^x^, and Le^y^; Jansson et al., 2006; Roussel et al., 2017).

Shiga toxin-producing *Escherichia coli* strains (STEC) can cause enterocolitis, bloody diarrhea, and, sometimes, a severe complication called hemolytic-uremic syndrome (HUS). Worldwide, STEC infects several million individuals annually, posing a growing threat to public health (Majowicz et al., 2014).

Shiga (Stx) toxins belong to the AB_5_ class of protein toxins (Fan et al., 2000). This group also includes pertussis toxin (Ptx), cholera toxin (Ctx), thermolabile enterotoxins produced by *E.coli* (LT-I and LT-II) and subtilase (SubAB; Paton et al., 2004; Johannes and Römer, 2010). The enzymatic activity responsible for cytotoxicity is located in the A subunit, which is often classified as a type II ribosome-inactivating protein (RIP) or ER-driven protein toxin (ERT, ER-routing protein toxin). The A subunit consists of the A1 and A2 fragments linked by a disulfide bridge. The pentamer of the B subunits forms a pore, containing the C-terminal fragment of the A subunit (Silva et al., 2017).

The pentamers of Shiga toxin B subunits bind GSLs of the globo series, showing a strong preference for the trisaccharide Gal*α*1 → 4Gal*β*1 → 4Glc. Stx2e (a subtype pathogenic for pigs) also uses Gb4Cer (GalNAcβ1 → 3Galα1 → 4Galβ1 → 4Glc) as a receptor as well as Gb5Cer with the structure Galβ1 → 3GalNAcβ1 → 3Galα1 → 4Galβ1 → 4Glc and GalNAcα1 → 3GalNAcβ1 → 3Galα1 → 4Galβ1 → 4Glcβ1 → 1Cer also known as the Forssman antigen (Gallegos et al., 2012; Detzner et al., 2021). Recently, it was shown that Shiga toxin can also bind Galα1 → 4Gal structures on N-glycans and use them as functional receptors (Szymczak-Kulus et al., 2021). Severe complications of STEC infection in humans result from Shiga toxin-mediated damage to the endothelium. This triggers release of proinflammatory cytokines, including tumor necrosis factor alpha (TNF-α), which inadvertently exacerbates the cytotoxicity by enhancing the synthesis of Gb3 in the endothelium, creating a vicious circle (Foster et al., 2000).

### Helicobacter pylori

*Helicobacter pylori* is the best-known pathogen of the human gastrointestinal tract, with half of the human population being infected. *Helicobacter pylori* colonizes the gastric mucosa. Prolonged infection may increase the risk of gastric cancer (Díaz et al., 2018). One of the *H. pylori* adhesins BabA binds Le^b^ from group O individuals fivefold greater than Le^b^ from group A individuals (Anstee, 2010; Benktander et al., 2012). In A and B blood type individuals, these glycotopes are hidden under the α1,3-linked residues of GalNAc or Gal, respectively, and BabA binds A and B antigens only weakly. Gastric mucosa of type O individuals is more susceptible to the binding of adhesin (Lingwood, 1999). During chronic infections, *H. pylori* upregulates the host *β3GnT5* gene expression, which leads to increased synthesis of the Le^x^ structure. In addition, the pathogen produces SabA, which is an adhesin that binds Le^x^ and Le^a^ glycoproteins (Kościelak, 2012; Jin et al., 2018). Both adhesins belong to the Helicobacter Outer Membrane Proteins family (HOPs), which also includes LabA. LabA recognizes the GalNAcβ1 → 4GlcNac structure, which is characteristic of the gastric mucosa glycoproteins. The HOP protein family have autotransporter-like architecture. The BabA and SabA adhesion ectodomains have similar topologies: 7 α-helices (4 + 3 system). In addition, BabA has a four-twisted β-sheet structure that comprises the binding site for the Le^b^ antigen (Moonens and Remaut, 2017). Results published by Teneberg and co-workers show that lactotetraosylceramide and lactosylceramide may also act as receptors for *H. pylori* (Teneberg et al., 1997, 2002; Angström et al., 1998; Roche et al., 2007).

### Pseudomonas aeruginosa

*Pseudomonas aeruginosa* is a pathogen that can attack the cell in a variety of ways. Factors contributing to bacterial invasion include GSLs, which affect the adhesion of *P. aeruginosa* to non-phagocytic cells. LecA, a homotetrameric galactophilic lectin located on the outer bacterial membrane binds GSLs (Kuhaudomlarp et al., 2020). Interaction of LecA with Gb3 produces large monolayer vesicles in the membrane enabling bacteria to enter the host cell (Blanchard et al., 2008; Kuhaudomlarp et al., 2020). This mechanism has been termed the “lipid zipper” and it depends on the composition of the lipid bilayer (Siukstaite et al., 2021). Cholesterol in lipid rafts stabilizes the Lec-A induced domains, and, with a sufficiently high density of Gb3, triggers a lipid zipper resulting in cell invasion. Blocking Lec-A/Gb3 interaction results in reduced invasion of *Pseudomonas aeruginosa*, and is a promising strategy for drug development (Eierhoff et al., 2014).

### Vibrio cholerae

*Vibrio cholerae* is the causative agent of cholera, a life-threatening diarrhoeal disease. The bacteria colonize the human small intestine and secrete their major virulence factor called cholera toxin (CT; Ali et al., 2016; Deen et al., 2020). CT is a member of the AB_5_ group of bacterial toxins, composed of one catalytically active A subunit (responsible for the cytotoxicity) and a homopentamer of the receptor-binding B subunits (Bharati and Ganguly, 2011). Originally, it was believed that the B subunit binds GM1 on the apical surface of intestinal epithelial cells. However, the role of GM1 is controversial because GM1 is rare in the human gastrointestinal tract (Holmgren et al., 1975; Wands et al., 2015). Recent studies suggest that fucosylated blood group antigens (e.g., Le^x^) may function as secondary receptors important for cellular uptake of the toxin and toxicity, but the mechanism remains unclear (Heim et al., 2019; Patry et al., 2019).

## Interaction between viruses and host GSLs

### HCMV

Human cytomegalovirus (HCMV) is a double-stranded linear DNA β-herpesvirus that typically causes a mild respiratory illness. The mechanism by which the virus infects cells is not fully understood. The infection consists of many stages and involves glycoconjugates. Heparin sulfate on the cell membrane was proposed to initiate binding of the virus. Most molecules reported in the literature as HCMV receptors are glycoproteins (Fernández-Moreno et al., 2021). However, HCMV shows a binding reactivity to SGGLs (sulfated glucuronyl glycosphingolipids), especially to SGLPG (sulfated glucuronyl lactosaminylparagloboside). Inhibition of SGGLs affects both the expression of the IE gene and the plaque formation by HCMV, indicating that the binding of HCMV to the sulfated carbohydrate epitope in SGGL plays an important role in the initial stages of infection. Notably, HCMV binds more strongly to SGLPG than to SGPG. Also, SGLPG had a stronger inhibitory effect than SGPG on HCMV infection. The lactosamine repeats (-3Galβ1 → 4GlcNAc1-)_2_ of SGLPG, in addition to the 3-sulfated glucuronyl moiety, may be important for recognition by HCMV. Lactosamine repeats could also play a role in preferential binding of HCMV to nLc_6_Cer compared to nLc_4_Cer (Ogawa-Goto et al., 1998).

### HIV

The genome of HIV comprises two plus (+) sense single RNA strands (Frankel and Young, 1998). The virus contains viral p17 matrix protein (MA), integrase (IN), and the viral protein R (Vpr) helping with viral RNA reverse transcription into double-stranded complementary DNA (cDNA) in the host cell cytoplasm following transport to the cell nucleus where it is integrated into the host cell genome; an envelope as well as a protein core (Lingwood, 2011).

The role of GSLs as receptors in HIV infection is complex. The viral adhesive protein gp120 forms a highly glycosylated trimeric complex on the viral membrane. The first GSL identified as a receptor for the gp120 protein was galactosylceramide and its sulfatide. Other receptors include GM3, GD3, and Gb3. Notably, gp120 shows binding preferences for different GSLs depending on the strain (GM3 for R5X4 HIV, Gb3 for HIV X4; Viard et al., 2003; Puryear and Gummuluru, 2012). The GSL binding site and the chemokine receptor are in the same loop (V3). The cholesterol/GSL ratio in lipid rafts may play a role in HIV infection. Densely packed lipids hinder the initial interaction of the viral protein with the receptor on the host cell (Liao et al., 2001). One of the viral proteins, called Nef, leads to downregulation of CD4 by directing it to a degradation pathway associated with the endoplasmic reticulum (Binette et al., 2007). Gp120 binding to GalCer triggers events leading to infection of CD4-negative cells. Modification of the lipid moiety in GalCer affects binding of the virus to CD4 +/− cells with varying degrees of inhibition. Gp120-GSL interactions may be influenced by the octamer of a conservative peptide from the V3 loop. The GSL binding site is at the center of the chemokine receptor binding in the gp120 protein (Liao et al., 2001). Lund and co-workers showed that a soluble Gb3 (adamantylGb3) analogue may bind gp120 and thus inhibit viral infection *in vitro*. These inhibition properties were tested for HIV X4, R5 strains, and resistant HIV-1 strains. A similar effect was demonstrated for other Gb3 analogues. Inhibition of infection may be due to blockage of the chemokine receptor binding site(Lund et al., 2006; Lingwood, 2011). An inverse relationship between the amount of Gb3 and HIV infection was shown for individuals with different genotypes in the P1PK blood group system. Lymphocytes lacking Gb3 (the null genotype called p) were the most sensitive, while T cells from the P1^k^ individuals were the most resistant (Lund et al., 2009).

### Human Parvovirus B19

The *Parvoviridae* is a single-stranded DNA small icosahedral viral family divided into *Parvovirinae* and *Densovirinae* subfamilies which can infect mammalian and invertebrate hosts, respectively. These subfamilies are further divided into five genera. Parvovirus B19 is classified as Erythrovirus (Thompson et al., 2019).

Human Parvovirus B19 was identified in the serum of an infected patient in 1975 (Brown and Young, 1995). Infection with the B19 parvovirus can cause erythema infectiosum (the Fifth disease), but also arthropathy (joint disease), fetal loss, and anemia. The main cause of anemia following B19 infection is binding of viral particles to erythroid precursors which leads to their destruction. Globoside (Gb4) was identified as the main receptor necessary for parvovirus B19 binding, but not competent for viral particles entry into host cells. For this, B19 requires another receptor: α5β1 integrin. However, this protein co-receptor is not present on mature red blood cells, which highly express Gb4, so B19 can bind but not enter mature red cells (Weigel-Kelley et al., 2003; Bieri and Ros, 2019; Thompson et al., 2019; Bieri et al., 2021). In addition to erythrocytes, B19 can infect several immune cells, including macrophages, B cells, T cells and follicular dendritic cells (Takahashi et al., 1998). Ku80 may play a role as a co-receptor, which mediates binding and entry of the virus (Munakata et al., 2005). Extensive studies on the role of Gb4 and other P1PK blood group system antigens in parvovirus B19 entry revealed that only cells from individuals with p phenotype are not susceptible to the virus. That finding gave rise to the hypothesis that the minimum glycan moiety for virus binding is GluNAc/GalNAcβ1→3Gal structure (Taube et al., 2010).

### Noroviruses

Caliciviruses are family of a small, single-stranded positive-sense RNA, non-enveloped and icosahedral viruses. The family consists of five major genera: Norovirus (humans, pigs and mice; Cao et al., 2007; Caddy et al., 2014), Sapovirus (human and pigs), Vesivirus (cats), Lagovirus (rabbits) and Nebovirus (cattle; Stuart and Brown, 2007; Kim et al., 2014). This viral family requires glycans for attachment and host cell entry.

Noroviruses infect human, bovine, and canine gastrointestinal epithelia, as well as RHDV (Rabbit Hemorrhagic Disease Virus), which target hepatocytes. All utilize human blood group-associated antigens (HBGAs) as cell-surface receptors (Huang et al., 2003; Hutson et al., 2003). On the other hand, GI-specific murine norovirus (MNV) and porcine sapovirus bind sialic acids mostly on O-glycans and some gangliosides (Ossiboff et al., 2007; Taube et al., 2010; Haga et al., 2016).

The activity of FUT genes in relation to expression of HBGA (secretor-type Fucα1→2Gal modifications) and levels of Lewis antigens (Fucα1→3/4GlcNAc) are associated with norovirus infection (Nasir et al., 2012; Almand et al., 2017; Someya, 2022). Localization of P2 (the most variable region of the P protein containing the carbohydrate-binding motif) dimers on the viral particle surface has prompted intensive research because of potential interactions with branches of glycans. The universality of these epitopes among mammalian species and the potential for emergence of new P2 variants raise concerns about interspecies transmission (Chassaing et al., 2020).

### Polyomaviruses

Polyomaviruses constitute a small family of double-stranded DNA, non-enveloped icosahedral viruses. The family of *Polyomaviridae* is divided into four genera: α-polyomavirus, β-polyomavirus, γ-polyomavirus, and δ-polyomavirus.

In humans, polyomavirus infections are common but mostly asymptomatic. They can be dangerous for immuno-compromised patients, in whom the infection symptoms may be severe and possibly lead to neoplasms and cancer (Neu et al., 2009; Maginnis et al., 2015).

The outer capsid of polyomavirus consists of pentamers of the VP1 coat protein with the glycan (particularly sialoglycan)-binding domain in most species (O’Hara et al., 2014).

Terminal NeuAcα2→3Galβ1→3GalNAc moiety is recognized by mouse polyomavirus (MPyV) and can be found in both gangliosides (e.g., GD1a) and glycoproteins (O-linked), although the contribution of these receptors in natural infections is still being examined (Tsai et al., 2003). Site-specific mutation at position 91 in VP1 allows binding the receptors with an additional NeuAc in the structure NeuAcα2→3Galβ1→3(NeuAcα2→6)GalNAc, which in some studies is associated with a decreased tumorigenic phenotype (O’Hara et al., 2014). BKV and JCV are well-recognized human polyomaviruses, identified in the same year. Their names were created from the initials of the patients from whom they were isolated (Gardner et al., 1971; Padgett et al., 1971). Both BKV and JCV polyomaviruses (HPyV-1 and HPyV-2, respectively) bind NeuAcα2→3Gal and NeuAcβ2→6Gal structures present in glycans of various ganglioside and glycoproteins from cells and tissues infected by these viruses (urogenital tissue, lymphocytes, renal cells, and astrocytes; Ströh et al., 2015). Merkel Cell Polyomavirus (MCPyV) is a new polyomavirus isolated from aggressive skin cancer, Merkel cell carcinoma (MCC; Gaynor et al., 2007). In recent reports, it was shown that only GT1b, but not GD1a or GD1b bind to MCPyV VP1 protein, suggesting that both α2,3-linked terminal and α2,8-linked internal sialic acids of GT1b are crucial for the interaction (Erickson et al., 2009). Another member of the family, SV40 virus is highly specific for the GM1 ganglioside, with NeuGc instead of NeuAc, but the interaction is relatively weak. Studies have shown that during the attachment viral molecules interact not only with GM1 but also with co-receptor the class I major histocompatibility complex (MHC-I) proteins and entering to cells in a caveolin-dependent or -independent manner (Pelkmans et al., 2001; Damm et al., 2005). Recognition of terminal NeuGc prevents SV40 infection of the human host (Neu et al., 2008).

### Rotaviruses

The *Reoviridae* (divided into two subfamilies: *Sedoreovirinae* and *Spinareovirinae*) are double-stranded RNA viruses, with complex, multilayered and icosahedral capsids. The most studied genera are Rotavirus (*Sedoreovirinae* subfamily) and Orthoreovirus (also named ‘reovirus,’ *Spinareovirinae* subfamily), which consist of large spike proteins projecting fivefold from the icosahedral (Settembre et al., 2011). The terminal domains of those spike proteins VP8 in rotavirus (Taube et al., 2010) and δ1 (Reiter et al., 2011) bind glycans (Sun et al., 2016).

Infections with rotaviruses (family *Reoviridae*) are the main cause of diarrhoea in young mammals. Rotaviral infections cause over 500,000 deaths in developing countries and are a frequent cause of childhood morbidity in industrialized countries. Moreover, infections result in significant losses in agriculture (diarrhoea in calf, pig, and poultry production). The infection involves two steps: viral recognition of and binding to the villus tip cells of the small intestine. Receptor analogs may inhibit the binding to host cells (Arias et al., 2008; Mukherjee and Chawla-Sarkar, 2011; Thompson et al., 2019). Human rotavirus (RV) infections are mostly caused by groups A and, to a lesser extent, C of RVs. Group A includes two types of strains: neuraminidase (NA) - sensitive and NA – insensitive, which is determined by the binding affinity of VP8* domain to terminal sialic acid moieties on the host cell surface carbohydrates (Delorme et al., 2001; Haselhorst et al., 2009; Kim et al., 2017). HBGAs, in particular A, H, and Lewis (Le^b^) antigens, appear to be the main receptors. Some studies suggest that secretor individuals are more susceptible to RV infections than nonsecretors. In the case of some RV strains (P[4], P[8]), nonsecretors develop neutralizing antibodies. Suggesting that they may be affected by those strains (Ayouni et al., 2015; Rodríguez-Díaz et al., 2017). It has been hypothesized that the higher frequency of infections among individuals with blood group A may signify evolution of RV in response to receptor changes in the host. These changes (e.g., αGal-to-GalNAc binding switch) have occurred under selection pressure in many animal species (Zhao et al., 2020).

### Fungal ligands binding GSLs

Fungal infections are a global health problem, with increasing incidence worldwide, particularly in patients with immune dysfunctions. It is estimated that over 1 billion people may be affected by fungal infections, including the most commonly detected aspergillosis (caused by *Aspergillus* spp.) and candidiasis (as a result of *Candida* spp. invasion), with an estimated mortality of 1 million annually (Lass-Flörl et al., 2021). Fungal pathogens bind many receptors, including glycosphingolipids. The most significant and the best described GSL receptor is lactosylceramide (Gal*β*1 → 4Glcβ-Cer) which is bound by the most ubiquitous fungi, such as *Cryptococcus neoformans*, *Candida albicans*, *Saccharomyces cerevisiae* and yeast phase of *Histoplasma capsulatum* and *Sporothrix schenckii* (Jimenez-Lucho et al., 1990). It was demonstrated that a clinically relevant fungus *C. neoformans*, responsible for cryptococcosis (May et al., 2016), strongly binds to LacCer-rich brain cells and cultured human glioma cells. Moreover, liposomes containing LacCer inhibited the fungi binding, while glucosylceramide and free lactose did not, confirming the involvement of LacCer in pathogen binding (Jimenez-Lucho et al., 1990). The binding of *C. neoformans* to LacCer is affected by the structure of the ceramide moiety. Semisynthetic lactosylceramide (named DL-dihydrolactocerebroside) with a short-chain fatty acid is not recognized by the fungal ligands. In addition, the terminal galactose residue of LacCer is essential for binding. Gal-terminated LacCer is crucial for *C. neoformans* binding because the LacCer precursor (glucosylceramide) and other lactosyl residue-containing neutral and acidic GSLs were not recognized by this pathogen (Jimenez-Lucho et al., 1990).

### *Candida* spp.

*Candida* spp. are a type of yeast that belong to Saccharomycetaceae family, which causes an array of infectious diseases collectively called candidiasis (Gow et al., 2011; Hani et al., 2015). Over 90% of infections are caused by five fungi: *C. albicans* (the major pathogen), *C. glabrata*, *C. tropicalis*, *C. parapsilosis* and *C. krusei* (Bongomin et al., 2017). These yeasts can recognize both non-GSL (stratherin, hydroxyapatite, fibrinogen and fibronectin; Johansson et al., 2000) and GSL receptors (LacCer, asialo-GM1, Le^a^ and H-active glycans; May et al., 1989; Jimenez-Lucho et al., 1990; Brassart et al., 1991; Yu et al., 1994; Johansson et al., 2000; Miyauchi et al., 2007). According to Jemeneza-Lucho LacCer is recognized by *C. albicans*, but these results were not confirmed in other studies (Yu et al., 1994). LacCer may be bound by *Candida* spp. or serve as a pattern recognition receptor to neutrophils in response to β-glucan, a component of *Candida* spp. cell wall (Sato et al., 2006). Generally, GSLs are recognized by lectin-like adhesins, whose specificity differs between strains and the recognition is influenced by growth conditions (Tosh and Douglas, 1992; Cameron and Douglas, 1996). Fucose-binding lectin is the best-studied *Candida* spp. adhesin. Oligosaccharides with terminal Fucα1 → 2Gal (O/H blood group antigen) were able to decrease *Candida* spp. adhesion to the epithelial cells, in contrast to internally fucosylated Lewis blood group antigens that did not show an inhibitory effect (Critchley and Douglas, 1987; Tosh and Douglas, 1992; Cameron and Douglas, 1996; Johansson et al., 2000). Other studies shed new light on the role of GSL-derived compounds in host membrane. Notably, GSLs in lipid rafts significantly influence the clearance of fungal pathogens by affecting interactions between *Candida* spp. and immune cells (in particular macrophages and neutrophils). Neutrophils are crucial immune cells involved in *Candida* spp. pathogenesis, because they are responsible for clearance of the pathogen. Moreover, macrophages contain LacCer in their own plasma membrane, raising the possibility of interaction with *Candida* spp. (Bryan et al., 2015; Tafesse et al., 2015).

It was demonstrated in several studies, that Lewis and ABO blood group phenotypes may be associated with susceptibility to *Candida* spp. The secretor/nonsecretor status (Lewis phenotypes) can influence vaginal candidiasis caused by C. albican*s* (Hilton et al., 1995; Kulkarni and Venkatesh, 2004). It was shown that Le(a-b-) phenotype was linked to recurrent vaginitis in women (Hilton et al., 1995), but when comparing Le(a + b-) with Le(a-b+) individuals there is no significant difference. Kulkarni and Venkatesh (2004) found correlation between *Candida* spp. and ABO phenotype and demonstrated that vaginal candidiasis occurs predominating in nonsecretors. It may be explained by the lack of ABO antigens in the body fluids of nonsecretors, which facilitates the attachment of *C. albicans* to the epithelial cells. Animal model studies support that secretor status may be protective against *C. albicans* vaginitis (Hurd and Domino, 2004; Domino et al., 2009), which may be also related to modulation of host–microbe interactions by estrogen, a key hormone upregulating FUT2 expression (responsible for secretor status; Fidel et al., 2000; Domino et al., 2009). Data on the role of ABO and Lewis secretor/nonsecretor status in oral candidiasis are conflicting (Aly et al., 1991; Ben-Aryeh et al., 1995). The proportion of nonsecretor status was significantly higher in patients with chronic hyperplastic candidiasis compared to the control subjects (Lamey et al., 1988). A small Indian study comparing ABO type and *Candida* spp. incidence in patients with duodenal ulcers (Singh et al., 1990) showed that *C. albicans* most frequently colonized patients with the O blood group, with higher incidence of nonsecretor status among the patients with peptic ulcers (Burford-Mason et al., 1993).

### Histoplasma capsulatum

*Histoplasma capsulatum* is a dimorphic fungus responsible for histoplasmosis, one of the most frequently occurring invasive fungal pulmonary infections (Gnat et al., 2021; Souza et al., 2021). This pathogen produces a cell surface 60 kDa heat shock protein (HSP60) which recognizes complement receptor 3 (CR3) on macrophages, leading to *H. capsulatum* HSP60 internalization. The interactions between immune cells and *H. capsulatum* depend on lipid microdomains on the cell surface, and the presence of sialylated GSLs, such as GM1, which is required for recruitment of CR3 into the cell membrane (Lin et al., 2010). Impaired adhesion in the absence of GM1 may be caused by two proposed mechanisms: (1) disorganization of lipid microdomains results in decreased association between macrophages and *H. capsulatum* or (2) GM1 may be a co-receptor in the initial steps of host-*H. capsulatum* interactions (Guimarães et al., 2019; Souza et al., 2021). In addition, LacCer, a key GSL in Src family tyrosine kinase Lyn-dependent signaling pathway, plays a crucial role in *H. capsulatum* pathogenesis by regulating cytoskeleton remodeling (Nakayama et al., 2013).

### Paracoccidioides brasiliensis

*Paracoccidioides brasiliensis* is a pathogenic dimorphic fungus that may cause paracoccidioidomycosis. According to Ywazaki et al. (2011) *P. brasiliensis* may bind GalCer, LacCer, CTH (Galα1 → 4Galα1 → 4Glcα1 → 1Cer), GD3, GM1 and GD1a. Another study showed that ganglioside GM1 localizes in lipid rafts of epithelial cells and may serve as receptor for this fungus (Maza et al., 2008). Also, GM3 occurring in lung fibroblast is involved in *P. brasiliensis* binding and/or infection (Ywazaki et al., 2011). Removal of *N*-acetylneuraminic acid from human alveolar cells resulted in decreased adhesion of *P. brasiliensis* conidia to these cells, suggesting that sialic acids may play a significant role in pathomechanism (González et al., 2008). Moreover, disruption of lipid rafts using cholesterol-sequestering agents (methyl-β-cyclodextrin, MβCD, or nystatin) hindered *P. brasiliensis* interaction with the cells (Maza et al., 2008). In addition, malate synthase can mediate *P. brasiliensis* interaction with mammalian cells because it acts as an adhesin (da Silva Neto et al., 2009).

### Pneumocystis jirovecii

*Pneumocystis jirovecii* is a pathogen responsible for opportunistic pneumonia in immunocompromised patients (Dellière et al., 2020) and children (Zakrzewska et al., 2021), with a relatively high mortality rate (Hahn et al., 2003). The main cell surface protein involved in host cell binding and internalization is β-glucan, a cell wall component containing 1,3-linked β-d-glucopyranosyl residues and variable amounts of 1,6-linked β-d-pyranosyl side chains, which may be recognized by LacCer in *S. cerevisiae* (Zimmerman et al., 1998). Hahn et al. (2003) found that blocking LacCer using a specific anti-LacCer antibody markedly decreased the release of macrophage inflammatory protein (MIP-2). Similar results were obtained using GSLs inhibitors (N-butyldeoxyno jirimycin and d-threo-1-phenyl-2-decanoylamino-3-morpholino-1-propanol-HCl), suggesting that LacCer may also play a significant role in inducing β-glucan-induced inflammatory signaling of *P. jirovecii* pathogenesis (Hahn et al., 2003; Souza et al., 2021).

## Discussion and future perspectives

Glycosphingolipids play several important roles in cell–cell interactions and communication in complex, multicellular organisms. The role of GSLs in mammals was studied by knock-outs of specific glycosyltransferases, mostly participating in initial steps of GSL biosynthesis (Yamashita et al., 1999, 2003; Kumagai et al., 2010; Wu et al., 2011; Allende and Proia, 2014). On the single-cell level, GSLs are involved in proliferation, apoptosis, endocytosis and cell migration, and thus they play a role in cancer progression (including breast, lung, colorectal and melanoma; Zhuo et al., 2018). This makes GSLs attractive targets for anti-tumor therapies (Kroll et al., 2020), including immunotherapy (Yu et al., 2020).

Autoimmunity induced by antigen mimicry, best described in *Campylobacter jejuni* infections, presents a unique challenge. Autoantibody responses against GM1 destroy host peripheral nerve components, leading to acute motor axonal neuropathy (AMAN) or acute motor-sensory axonal neuropathy (an axonal form of GBS, based on the absence/presence of sensory involvement). One GBS variant, Miller Fisher syndrome, is characterized by gait ataxia, areflexia, and acute ophthalmoplegia (Lee et al., 2004; Brezovska et al., 2011; Koike and Katsuno, 2021). Molecular mechanisms driving the development of autoantibodies and host complement activation remain incompletely understood.

The importance of GSLs as receptors is linked to their localization in lipid rafts in the plasma membrane. Networks of lipid rafts and other membrane elements (mainly membrane-associated proteins) influence host-pathogen interactions by affecting pathogen or toxin sequestration and cell entry. Shiga and cholera toxins enter the host cells *via* GSL receptors, but successful entry and cytotoxicity depend on multiple factors, including the composition of plasma membrane, organization of lipid and protein components, clustering of GSL receptors, and actin remodeling. Membrane microdomains called lipid rafts contain a special subset of proteins (e.g., G proteins) and lipids (cholesterol, GPI-anchored proteins, and glycolipids in the outer leaflet, and unsaturated phospholipids and caveolin in the inner leaflet). Many studies suggested that entry through lipid raft components helps pathogens evade host immune responses and lysosomal fusion. Disruption of lipid rafts (e.g., depletion of cholesterol) inhibits or stimulates signaling pathways. The ceramide moiety of GSLs also impacts their receptor functions. For example, Shiga toxins bind more strongly to Gb3 with long-chain fatty acid (C20-C24) compared to shorter ones (C12-C14). Additionally, Gb3 isoforms with saturated fatty acid chains show lower affinity to Stx compared to unsaturated chains. These elements of the receptor chemistry, distribution of receptors in the cell membrane as well as the configuration and number of receptor binding sites on the ligands determine whether the toxin can induce negative membrane curvature and tubular plasma membrane intussusception necessary for cellular uptake (Römer et al., 2007, 2010; Johannes and Römer, 2010; Pezeshkian et al., 2016; Johannes and Verhelst, 2021).

The mechanisms whereby pathogens and toxins engage GSLs to thrive in the host or induce cytotoxicity are more challenging to understand than the traditional protein–protein interaction paradigm (Kulkarni et al., 2021). The multiple examples of infections or pathologies involving GSLs point to untapped potential and call for unorthodox approaches in search of new treatments.

## Author contributions

AB, KM and RK wrote the manuscript. RK and MC reviewed and edited the manuscript. KM prepared the figures. All authors have read and agreed to the published version of the manuscript. All authors contributed to the article and approved the submitted version.

## Funding

This research was funded by the National Science Centre of Poland, OPUS project 2018/31/B/NZ6/01828 (MC).

## Conflict of interest

The authors declare that the research was conducted in the absence of any commercial or financial relationships that could be construed as a potential conflict of interest.

## Publisher’s note

All claims expressed in this article are solely those of the authors and do not necessarily represent those of their affiliated organizations, or those of the publisher, the editors and the reviewers. Any product that may be evaluated in this article, or claim that may be made by its manufacturer, is not guaranteed or endorsed by the publisher.

## Abbreviations

FUT: Fucosyltransferase
GalCer: Galactosylceramide
Gb3: Globotriaosylceramide
Gb4: Globotetraosylceramide
GgO_3_: Gangliotriaosylceramide
GgO_4_: Gangliotetraosylceramide
GlcCer: Glucosylceramide
GM1: Monosialotetrahexosylganglioside
HBGAs: Human Blood Group Associated Antigens
LPS: Lipopolysaccharide
SGGLs: Sulfated Glucuronyl Glycosphingolipids
SGLPG: Sulfated Glucuronyl Lactosaminylparagloboside
SGPG: Sulfated Glucuronyl Paragloboside
CTH: Ceramide Trihexoside
